# Parallel Mediating Effects of Sleep Quality, Psychological Distress, and Self‐Stigma in the Associations Between Long COVID Symptoms and Quality of Life Among Taiwanese Individuals With Mental Health Illness

**DOI:** 10.1002/brb3.70094

**Published:** 2024-10-14

**Authors:** Kun‐Chia Chang, Daniel Kwasi Ahorsu, Hsin‐Chi Tsai, Carol Strong, Nai‐Ying Ko, Jung‐Sheng Chen, Cheng‐Fang Yen, Servet Üztemur, Mark D. Griffiths, Chung‐Ying Lin

**Affiliations:** ^1^ Department of General Psychiatry, Jianan Psychiatric Center Ministry of Health and Welfare Tainan Taiwan; ^2^ Department of Psychiatry, National Cheng Kung University Hospital, College of Medicine National Cheng Kung University Tainan Taiwan; ^3^ Department of Special Education and Counselling The Education University of Hong Kong Tai Po New Territories Hong Kong; ^4^ Department of Psychiatry, School of Medicine Tzu Chi University Hualien Taiwan; ^5^ Department of Psychiatry Tzu‐Chi General Hospital Hualien Taiwan; ^6^ Department of Public Health, College of Medicine National Cheng Kung University Tainan Taiwan; ^7^ Department of Nursing, College of Medicine National Cheng Kung University Tainan Taiwan; ^8^ Department of Medical Research E‐Da Hospital, I‐Shou University Kaohsiung Taiwan; ^9^ Department of Psychiatry Kaohsiung Medical University Hospital Kaohsiung Taiwan; ^10^ Department of Psychiatry, School of Medicine, College of Medicine Kaohsiung Medical University Kaohsiung Taiwan; ^11^ College of Professional Studies National Pingtung University of Science and Technology Pingtung Taiwan; ^12^ Department of Turkish and Social Sciences Education, Faculty of Education Anadolu University Eskişehir Türkiye; ^13^ Psychology Department Nottingham Trent University Nottingham UK; ^14^ Institute of Allied Health Sciences, College of Medicine National Cheng Kung University Tainan Taiwan; ^15^ Biostatistics Consulting Center, National Cheng Kung University Hospital, College of Medicine National Cheng Kung University Tainan Taiwan; ^16^ Department of Occupational Therapy, College of Medicine National Cheng Kung University Tainan Taiwan

**Keywords:** COVID‐19, mental illness, psychological distress, quality of life, sleep, stigma

## Abstract

**Background:**

Long COVID symptoms (i.e., experiencing symptoms of COVID‐19 for 3 months post‐COVID‐19) affect individuals’ health and their quality of life (QoL). However, the pathways through which it does so are not fully known.

**Aim:**

The present study examined the mediating roles of sleep quality, psychological distress, and self‐stigma in the associations between long COVID symptoms and QoL among individuals with mental illness.

**Method:**

Individuals with mental illness (*n* = 333) were recruited from a psychiatric center in southern Taiwan to participate in the study. Data were collected regarding sleep quality, psychological distress, self‐stigma, and QoL. Independent *t*‐tests, Pearson correlations, and regression with Hayes’ Process Macro were used to compare groups, examine relationships, and parallel mediation models, respectively.

**Results:**

Participants with long COVID symptoms had significantly worse sleep quality, psychological distress, physical QoL, and psychological QoL compared to those without symptoms. There were significant relationships between sleep quality, psychological distress, self‐stigma, and QoL. Sleep quality significantly mediated the associations between long COVID symptoms and physical and social QoL. Psychological distress significantly mediated the associations between long COVID symptoms and all domains of QoL, but not self‐stigma.

**Conclusion:**

There are alternative pathways (e.g., sleep quality and psychological distress) through which long COVID symptoms may affect the QoL of individuals with mental illness. The findings suggest that individuals with long COVID symptoms have a higher chance of having poor QoL. Therefore, there may be the need for counseling and possible therapy for those who contract COVID‐19, especially among individuals who already have mental illness.

## Graphical abstract

There are alternative pathways (e.g., sleep quality and psychological distress) through which long COVID symptoms may associate with quality of life (QoL) among individuals with mental illness.

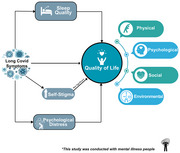

## 1 Introduction

The effect of coronavirus 2019 disease (COVID‐19) on individuals’ mental, physical, and/or social health has been reported in many studies (Andrade et al. 2022; Alimohamadi et al. 2020; Hossain, Sultana, and Purohit 2020; Ashwlayan et al. 2022; Kumar et al. 2021; Beyerstedt, Casaro, and Rangel 2021; Shen et al. 2023; Grant et al. 2020; Brooks‐Hay, Saunders, and Burman 2022; Mahlangu et al. 2022; Vicerra, De Pano, and Estanislao 2022; Kar, Kar, and Panda 2023; Addo 2020; Atashi et al. 2023; Vicerra 2022; Khankeh et al. 2022; Loades et al. 2020; Brooks et al. 2020). The novel severe acute respiratory syndrome coronavirus 2 (SARS‐CoV‐2) affects physical health by first attaching itself to the human body through the angiotensin‐converting enzyme 2 (ACE 2) receptor (Ashwlayan et al. 2022; Kumar et al. 2021). This consequently affects blood pressure and other multiple organ systems such as the nervous system, immune system, cardiovascular system, and endocrine system (Ashwlayan et al. 2022; Beyerstedt, Casaro, and Rangel 2021). These compromised systems affect basic life‐surviving functions such as breathing, especially among individuals with comorbidities including hypertension, diabetes mellitus, cardiovascular disease, and mental health (Ashwlayan et al. 2022).

The physiological effects of COVID‐19 are also manifested symptomatically. Most individuals affected by COVID‐19 exhibit symptoms of fever, cough, chest pain, dizziness, heart palpitations, fatigue/low energy, dyspnea, sleep problems, coughing up phlegm, and back pain (Alimohamadi et al. 2020; Shen et al. 2023; Grant et al. 2020). The introduction of preventive measures (e.g., quarantine and lockdowns) inadvertently led to other social (e.g., domestic violence and abuse, discrimination) (Brooks‐Hay, Saunders, and Burman 2022; Mahlangu et al. 2022; Vicerra, De Pano, and Estanislao 2022; Kar, Kar, and Panda 2023; Addo 2020) and mental health challenges (Hossain, Sultana, and Purohit 2020; Atashi et al. 2023; Vicerra 2022; Khankeh et al. 2022; Loades et al. 2020; Brooks et al. 2020), apart from the effects of COVID‐19 itself. These consequences had significant impacts on physical, psychological, social, and environmental quality of life (QoL) (Figueiredo et al. 2021; AlNujaidi et al. 2022; Hawlader et al. 2021) among those affected by COVID‐19, and possibly worse among individuals with mental illness because they may not be able to decipher or recognize the COVID‐19 symptoms compared to their healthy counterparts, thereby further worsening their QoL (Druss 2020; H. Yao, Chen, and Xu 2020; Chang et al. 2020; Chang et al. 2022). It is also known that some individuals do not recover from COVID‐19 (i.e., they experience “long COVID”) for an extended period of time.

There is no consensus on the definition of long COVID, which is also known as post‐COVID‐19 condition, post‐acute COVID‐19 syndrome, and post‐acute sequelae of COVID‐19 (PASC) (Davis et al. 2023; Subramanian et al. 2022; World Health Organization 2021; Lopez‐Leon et al. 2021; Davis et al. 2021). However, it involves a multisystem condition that comprises some of the symptoms of COVID‐19 lasting at least 2 months postinfection and cannot be explained by any other alternative diagnosis among individuals who have a probable or confirmed history of SARS CoV‐2 infection (Davis et al. 2023; Subramanian et al. 2022; World Health Organization 2021). However, the present study used a symptom duration of 1 month after detection of COVID‐19, as strongly advocated by individuals with lived experience of this condition (Sivan and Taylor 2020). These symptoms including fatigue, shortness of breath, and cognitive dysfunction impact everyday functioning (Davis et al. 2023; Subramanian et al. 2022; World Health Organization 2021). Surprisingly, long COVID can also occur among vaccinated individuals (Al‐Aly, Bowe, and Xie 2022; Byambasuren et al. 2023).

The deleterious impact of long COVID on the functioning and (ultimately) QoL of individuals has been much reported (Ceban et al. 2022; Fischer et al. 2022; Líška et al. 2022; Poudel et al. 2021; O' Mahony et al. 2022). Previous studies and reviews have reported that individuals with long COVID experience prolonged multisystem conditions and significant disability lasting several months (Ceban et al. 2022) with some even lasting more than 7 months post‐COVID‐19 (Davis et al. 2021; Kim et al. 2024). More specifically, long COVID affects individuals’ QoL through challenges to their physical health (e.g., fatigue, musculoskeletal and neurologic disorders) (Al‐Aly, Bowe, and Xie 2022; Ceban et al. 2022; Kim et al. 2024), psychological health (e.g., cognitive impairment, mental health disorder) (Al‐Aly, Bowe, and Xie 2022; Ceban et al. 2022; Kim et al. 2024), social relationships (Aghaei et al. 2022; Trihandini et al. 2023), and environmental health (e.g., recreation and leisure, home environment, access and quality of health and social care, freedom) (Dale et al. 2022; Mouratidis and Yiannakou 2022).

Individuals with long COVID symptoms have been found to have poor sleep quality (Fischer et al. 2022; Bourmistrova et al. 2022; Mekhael et al. 2022), psychological distress (i.e., depression, anxiety, and stress) (Fischer et al. 2022; Líška et al. 2022; O' Mahony et al. 2022; Bourmistrova et al. 2022), and stigma (Ballering, Olde Hartman, and Rosmalen 2021; Pantelic et al. 2022) with known associations between these variables and poor QoL (Ballering, Olde Hartman, and Rosmalen 2021; Scholz, Bierbauer, and Lüscher 2023; Orrù et al. 2021). Moreover, these associations are also experienced by individuals with mental illness (Yen et al. 2009; Saffari et al. 2023; Zheng et al. 2023), which suggests that sleep quality, psychological distress (i.e., depression, anxiety, and stress), and self‐stigma may be potential mediating factors in the association between long COVID symptoms and poor QoL. Furthermore, despite the purported effect of long COVID on different aspects of a person's QoL, it is not very clear how long COVID or its symptoms affect individuals’ QoL. There are several potential pathways (i.e., directly or indirectly—via other factors) through which long COVID symptoms may affect individuals’ QoL. Therefore, it is important to examine the pathways through which long COVID symptoms affect individuals’ QoL, especially as there are no previous studies that have examined the mediating roles of other factors in the association between long COVID symptoms and QoL.

Considering that long COVID symptoms significantly affect individuals’ health, functioning, and (consequently) physical, psychological, social, and environmental QoL, it is imperative to examine how long COVID symptoms significantly affect a person's physical, psychological, social, and environmental QoL. This is needed and particularly important among individuals with mental illness because (1) there are known associations between sleep quality, psychological distress (i.e., depression, anxiety, and stress), self‐stigma, and QoL (Yen et al. 2009; Saffari et al. 2023; Zheng et al. 2023), (2) they are a relatively unexamined group with respect to COVID‐19 (H. Yao, Chen, and Xu 2020), and (3) they are among the most vulnerable individuals in society (Murphy et al. 2021).

Therefore, the present study examined the mediating roles of sleep quality, psychological distress (i.e., depression, anxiety, and stress), and self‐stigma in the associations between long COVID symptoms and poor QoL taking into consideration that the participants are individuals with mental health illness. More specifically, the present study examined (1) differences between individuals with long COVID symptoms and those without in relation to the study's variables (i.e., sleep quality, psychological distress, self‐stigma, and QoL); (2) the associations between long COVID symptoms, sleep quality, psychological distress, self‐stigma, and QoL; and (3) the mediating roles of sleep quality, psychological distress, and self‐stigma in the association between long COVID symptoms and QoL (i.e., physical, psychological, social, and environmental QoL). It was therefore hypothesized that (1) there would be significant differences between individuals with or without long COVID symptoms in relation to the study's variables (i.e., sleep quality, psychological distress, self‐stigma, and QoL); (2) there would be significant associations between long COVID symptoms, sleep quality, psychological distress, self‐stigma, and QoL; and (3) sleep quality, psychological distress, and self‐stigma would be mediators in the association between long COVID symptoms and QoL (i.e., physical, psychological, social, and environmental QoL).

## 2 Methods

### 2.1 Setting, Participants, and Data Collection Procedure

A cross‐sectional study was initiated during the period that the Taiwan government eased its regulations in response to the COVID‐19 pandemic and then downgraded COVID‐19 to Category 4^1^ notifiable communicable disease. To facilitate protection of privacy for participants with mental health illnesses, they were recruited at their most convenient treatment site, namely, the Jianan Psychiatric Centre (JPC), therefore protecting their identities within their living communities. The JPC, located in Tainan, a central city of southern Taiwan, is the only national teaching psychiatric center in this area. Also, it is a core center for the psychiatric treatment network in southern Taiwan which indicates its capacity to provide mental health services to more than three million residents there. The JPC provides intensive rehabilitation programs, day care programs, outpatient services, and home care as a whole continuous treatment supply chain for thousands of individuals with mental health illnesses. The JPC also provides oral antiviral drugs for individuals with mental health illnesses who test positive with the SARS‐CoV‐2 rapid test after the symptoms’ evaluation process. The data collection took place between October 18, 2022 and May 18, 2023, with eligible participants being recruited from the outpatient units, day care, and intensive rehabilitation programs at JPC.

The inclusion criteria were as follows: (1) having at least one major diagnosis of mental health disorder as diagnosed by one of the psychiatrists using the DSM‐5 (fifth edition of the *Diagnostic and Statistical Manual of Mental Disorders*) criteria; (2) having sufficient mental capacity to provide consent and complete the assessments; (3) admission to the daycare or intensive rehabilitation programs or having regular follow‐ups at the outpatient unit, which indicate that the patient had relatively stable condition (e.g., psychiatric symptoms are residual or not active); (4) being more than 20 years old; (5) not being currently affected by COVID‐19 infection or being at least 4 weeks after acute COVID‐19 infection was confirmed; and (6) having SARS‐CoV‐2 rapid test negativity at the time of study entry. The exclusion criteria were as follows: (1) having a severe and unstable medical disease or a history of neurological disease, and (2) having a history of head injury.

A total of 333 participants took part in the study. The present study protocol was approved by the Institutional Review Board (IRB) of Jianan Psychiatric Center, Ministry of Health and Welfare (IRB numbers: 22‐008). All procedures contributing to the study complied with the ethical standards of the relevant national and institutional committees on human experimentation and as enshrined in the Helsinki Declaration of 1975, as revised in 2013.

### 2.2 Measures

#### 2.2.1 Demographics and Clinical Characteristics

The demographics of the participants were asked using a self‐reported background information sheet, which included their age (in years), sex (male or female), marital status (married, single, or other), and number of years in formal education. Regarding their clinical characteristics, a research assistant checked their medical records and recorded their physiological diseases, diagnoses of mental health illnesses, and any post‐acute sequelae of SARS‐CoV‐2 infection (that is, long COVID‐19 with symptoms duration > 1 month after detection of COVID‐19). The physician was in contact with the hospital to ensure that participants were diagnosed with long COVID at the time of assessment.

#### 2.2.2 Sleep Quality

The sleep quality of participants was assessed using the 19‐item self‐report Pittsburgh Sleep Quality Index (PSQI). The 19 items are responded to using two response‐type scales (i.e., a four‐point Likert scale or a direct answer). The responses are then calculated into seven components, with each score between 0 and 3, where a higher score indicates a poorer level of sleep quality. The seven component scores were summed up to indicate the overall sleep quality (Buysse et al. 1989; Carpenter and Andrykowski 1998). Prior empirical evidence has shown that the PSQI, including its Chinese version, have good psychometric properties (Wang et al. 2022; Zhu et al. 2018; Tsai et al. 2005). In the present study, the internal consistency of the PSQI was acceptable (*α* = 0.61).

#### 2.2.3 Psychological Distress

The psychological distress of participants was assessed using the 21‐item self‐report Depression, Anxiety and Stress Scale (DASS‐21). The 21 items are responded to using a four‐point Likert scale between 0 and 3, with a higher score indicating a greater level of psychological distress. The 21‐item scores are summed up to indicate the overall psychological distress (Lovibond and Lovibond 1995). Prior empirical evidence has shown that the DASS‐21, including its Chinese version, has good psychometric properties (Chen et al. 2023; Cao et al. 2023.). In the present study, the internal consistency of the DASS‐21 was excellent (*α* = 0.96).

#### 2.2.4 Self‐Stigma

The self‐stigma of participants (regarding the identity of having a mental health illness) was assessed using the nine‐item self‐report Self‐Stigma Scale‐Short (SSS‐S). The nine items are responded to using a four‐point Likert scale between 1 and 4, with a higher score indicating a greater level of self‐stigma. The nine‐item scores are summed up to indicate the overall self‐stigma (Mak and Cheung 2010). Prior empirical evidence has shown that the SSS‐S, including its Chinese version, has good psychometric properties (Mak and Cheung 2010; Wu et al. 2015). In the present study, the internal consistency of the SSS‐S was excellent (*α* = 0.94).

#### 2.2.5 Quality of Life

The QoL of participants was assessed using the 28‐item self‐report World Health Organization Quality of Life Questionnaire‐Brief version (WHOQOL‐BREF). The 28 items are responded to using a five‐point Likert scale between 1 and 5, with a higher score indicating a better QoL. The first two items in the WHOQOL‐BREF ask about general health and were not used in the present study's data analysis. The remaining 26 WHOQOL‐BREF items are distributed into four domains: physical QoL (seven items), psychological QoL (six items), social QoL (four items), and environmental QoL (nine items). Item scores of each domain are summed up to indicate the QoL in each dimension (THE WHOQOL GROUP 1998). Prior empirical evidence has shown that the WHOQOL‐BREF, including its Chinese version, have good psychometric properties (G. Yao et al. 2002; Hwang et al. 2003). In the present study, the internal consistencies of the WHOQOL‐BREF subscales were very good (*α* = 0.81 [physical QoL], 0.84 [psychological QoL], 0.83 [social QoL], and 0.90 [environmental QoL]).

### 2.3 Data Analysis

The participants’ demographics, clinical characteristics, and scores of the psychometric scales (i.e., PSQI, DASS‐21, SSS‐S, and WHOQOL‐BREF) were analyzed using descriptive statistics (means for continuous data and frequencies for categorical data). Independent *t*‐tests (for continuous data) and χ^2^ tests (for categorical data) were used to compare groups (i.e., the long COVID group and the non‐long COVID group) in their demographics, clinical characteristics, and scores on the scales. Pearson correlations were conducted to examine the relationships between scale scores. Lastly, Hayes’ Process Macro was used to perform the parallel mediation models (using Model 4 in Hayes’ Process Macro). Four parallel mediation models were performed using the same set of the independent variable (i.e., long COVID group or not), mediators (i.e., sleep quality, psychological distress, and self‐stigma), and control variables (i.e., age and sex) but different dependent variables (i.e., physical, psychological, social, and environmental QoL). The mediated effects were examined using the bootstrapping method, where 5000 bootstrapping resamples were generated. The significance was set at the following two conditions: (1) *p* < 0.05 or (2) the bootstrapping upper limit confidence interval (ULCI) and lower limit confidence interval (LLCI) do not cover 0. All the analyses were conducted using the SAS version 9.4.

## 3 Results

Among the 333 participants, 79 had at least one long COVID symptom (23.7%). Out of the 333 participants, 211 were diagnosed with schizophrenia or other psychotic disorders (63.4%) and 55 were diagnosed with bipolar disorder (16.5%), with nearly 70% (*N* = 233) receiving antipsychotics (mostly second‐generation antipsychotics). However, there were no significant differences between the diseases or the medication categories. Detailed information on all the long COVID symptoms is reported in Table 1. In brief, the most common symptom was coughing (62.03%), followed by fatigue (31.65%) and brain fog (25.32%). When comparing the age, sex, physiological disease, marital status, diagnosis, and educational year between the two groups (i.e., long COVID symptom group vs. no symptom group), there were no significant differences (*p*‐values ranging between 0.059 and 0.753). Regarding the scale scores (i.e., PSQI, DASS‐21, SSS‐S, and WHOQOL‐BREF), the long COVID symptom group had significantly poorer sleep quality (mean score = 10.18 [SD = 4.02] vs. 8.79 [SD = 4.07]; *p* = 0.009), greater psychological distress (mean score = 15.80 [SD = 13.87] vs. 12.15 [SD = 12.25]; *p* = 0.026), worse physical QoL (mean score = 12.85 [SD = 2.60] vs. 13.77 [SD = 2.93]; *p* = 0.014), and worse psychological QoL (mean score = 11.83 [SD = 3.20] vs. 12.78 [SD = 3.26]; *p* = 0.023) (Table 2). Moreover, compared to participants with other long COVID symptoms, those with respiratory symptoms had significantly (1) poorer sleep quality (mean score = 13.92 [SD = 3.52] vs. 9.43 [SD = 3.70]; *p* < 0.001); (2) greater psychological distress (mean score = 28.92 [SD = 18.35] vs. 13.21 [SD = 11.29]; *p* = 0.010); and (3) poorer physical QoL (mean score = 10.51 [SD = 3.02] vs. 13.32 [SD = 2.26]; *p* < 0.001), psychological QoL (mean score = 9.49 [SD = 3.57] vs. 12.30 [SD = 2.93]; *p* = 0.003), and social QoL (mean score = 10.23 [SD = 2.89] vs. 12.73 [SD = 2.99]; *p* = 0.007) (Table 3).

**TABLE 1:** Frequencies of the long COVID symptoms (*N* = 79).

	Long COVID symptoms
	*n* (%)
Fatigue	25 (31.65)
Short of breath	13 (16.46)
Depressed	14 (17.72)
Chest pain	4 (5.06)
Heart palpitations	2 (2.53)
Muscle aches	10 (12.66)
Joint pain	11 (13.92)
Cough	49 (62.03)
Headache	18 (22.78)
Brain frog	20 (25.32)
Olfactory problems	4 (5.06)
Taste problems	7 (8.86)
Number of long COVID symptoms	
1	29 (36.71)
2	24 (30.38)
3	14 (17.72)
4	5 (6.33)
≥ 5	7 (8.86)

**TABLE 2:** Independent *t*‐tests and χ^2^ tests (or Fisher's exact test) between two groups (i.e., long COVID symptoms vs. no long COVID symptoms).

	Long COVID symptoms (*N* = 79)	No symptoms (*N* = 254)	
	*M* (SD) or *n* (%)	*M* (SD) or *n* (%)	*p* value
Age (in years)	47.04 (10.36)	49.52 (10.08)	0.059
Sex (males)	50 (63.29)	154 (60.63)	0.672
Hypertension	17 (21.52)	43 (16.93)	0.354
Endocarditis	1 (1.27)	0 (0.00)	0.237^a^
Diabetes	12 (15.19)	35 (13.78)	0.753
Stroke	1 (1.27)	0 (0.00)	0.237^a^
HBV	8 (10.13)	12 (4.72)	0.101^a^
HCV	5 (6.33)	9 (3.54)	0.334 ^a^
Cirrhosis	1 (1.27)	0 (0.00)	0.238^a^
Pancreatitis	1 (1.27)	0 (0.00)	0.237^a^
Cellulitis	0 (0.00)	1 (0.39)	1.000^a^
HIV	1 (1.27)	2 (0.79)	0.557^a^
Marital status			0.120
Married	21 (26.58)	46 (18.11)	
Single	43 (54.43)	170 (66.93)	
Other	15 (18.99)	38 (14.96)	
Diagnosis			0.250
Schizophrenia	46 (58.23)	165 (64.96)	
Bipolar	16 (20.25)	39 (15.35)	
Major depression	10 (12.66)	17 (6.69)	
Substance use	5 (6.33)	28 (11.02)	
Other	2 (2.53)	5 (1.97)	
Educational year	11.06 (3.11)	11.45 (2.74)	0.284
Sleep quality	10.18 (4.02)	8.79 (4.07)	0.009
Self‐stigma	2.17 (0.76)	2.13 (0.75)	0.710
Psychological distress	15.80 (13.87)	12.15 (12.25)	0.026
Physical QoL	12.85 (2.60)	13.77 (2.93)	0.014
Psychological QoL	11.83 (3.20)	12.78 (3.26)	0.023
Social QoL	12.32 (3.10)	13.00 (3.11)	0.089
Environmental QoL	12.61 (2.68)	13.27 (3.06)	0.085

*Note*: Sleep quality was assessed using the Pittsburgh Sleep Quality Index; quality of life (QoL) was assessed using the World Health Organization Quality of Life Questionnaire‐Brief version; self‐stigma was assessed using the Self‐Stigma Scale‐Short; and psychological distress was assessed using the Depression, Anxiety and Stress Scale‐21.

^a^ Fisher's exact test was used because > 20% cells having the expected values are smaller than 5.

**TABLE 3:** Independent *t*‐tests and χ^2^ tests (or Fisher's exact test) between groups with different long COVID symptoms (*N* = 79).

	Respiratory symptoms (*N* = 13)	Other symptoms (*N* = 66)	
	*M* (SD) or *n* (%)	*M* (SD) or *n* (%)	*p* value
Age (in years)	46.85 (13.99)	47.08 (9.63)	0.942
Sex (males)	8 (61.54)	42 (63.64)	1.000
Hypertension	5 (38.46)	12 (18.18)	0.139^a^
Endocarditis	1 (7.69)	0 (0.00)	0.165^a^
Diabetes	1 (7.69)	11 (16.67)	0.679^a^
Stroke	1 (7.69)	0 (0.00)	0.165^a^
HBV	1 (7.69)	7 (10.61)	1.000^a^
HCV	0 (0.00)	5 (7.58)	0.584^a^
Cirrhosis	0 (0.00)	1 (1.52)	1.000^a^
Pancreatitis	0 (0.00)	1 (1.52)	1.000^a^
Cellulitis	0 (0.00)	0 (0.00)	—
HIV	0 (0.00)	1 (1.52)	1.000^a^
Marital status			0.053^a^
Married	7 (53.85)	14 (21.21)	
Single	4 (30.77)	39 (59.09)	
Other	2 (15.38)	13 (19.70)	
Diagnosis			0.006^a^
Schizophrenia	3 (23.08)	43 (65.15)	
Bipolar	3 (23.08)	13 (19.70)	
Major depression	5 (38.46)	5 (7.58)	
Substance use	2 (15.38)	3 (4.55)	
Other	0 (0.00)	2 (3.03)	
Educational year	9.77 (3.70)	11.32 (2.94)	0.101
Sleep quality	13.92 (3.52)	9.43 (3.70)	< 0.001
Self‐stigma	1.82 (0.94)	2.24 (0.71)	0.070
Psychological distress	28.92 (18.35)	13.21 (11.29)	0.010
Physical QoL	10.51 (3.02)	13.32 (2.26)	< 0.001
Psychological QoL	9.49 (3.57)	12.30 (2.93)	0.003
Social QoL	10.23 (2.89)	12.73 (2.99)	0.007
Environmental QoL	11.93 (3.43)	12.74 (2.52)	0.323

*Note*: Sleep quality was assessed using the Pittsburgh Sleep Quality Index; quality of life (QoL) was assessed using the World Health Organization Quality of Life Questionnaire‐Brief version; self‐stigma was assessed using the Self‐Stigma Scale‐Short; and psychological distress was assessed using the Depression, Anxiety and Stress Scale‐21.

^a^ Fisher's exact test was used because > 20% cells having the expected values are smaller than 5.

Pearson correlation coefficients shown in Table 4 further indicate that all the scores on the study scales were significantly related to each other (all *p*‐values < 0.001). More specifically, poorer sleep quality was related to worse QoL in all domains (*r* = −0.49 to − 0.25); stronger self‐stigma was related to worse QoL in all domains (*r* = −0.38 to − 0.31); and greater psychological distress was related to QoL in all domains (*r* = −0.47 to − 0.61). Moreover, poor sleep quality, self‐stigma, and psychological distress were positively related to each other (*r* = 0.19 to 0.48). In addition, Spearman correlation coefficients showed that a greater number of symptoms were associated with (i) poorer sleep quality (*r* = 0.19; *p* < 0.001); (ii) poorer physical QoL (*r* = −0.16; *p* = 0.004), psychological QoL (*r* = −0.14; *p* = 0.013), and social QoL (*r* = −0.12; *p* = 0.027); and (iii) greater level of psychological distress (*r* = 0.16; *p* = 0.004).

**TABLE 4:** Correlations between sleep quality, quality of life (QoL), self‐stigma, and psychological distress (distress) (*N* = 333).

	Sleep quality	Physical QoL	Psychological QoL	Social QoL	Environmenta QoL	Self‐stigma	Distress	Number of symptoms^a^
Sleep quality	—	− 0.49 (< 0.001)	− 0.35 (< 0.001)	− 0.31 (< 0.001)	− 0.25 (< 0.001)	0.19 (< 0.001)	0.47 (< 0.001)	0.19 (< 0.001)
Physical QoL		—	0.77 (< 0.001)	0.64 (< 0.001)	0.72 (< 0.001)	− 0.36 (< 0.001)	− 0.61 (< 0.001)	− 0.16 (0.004)
Psychological QoL			—	0.69 (< 0.001)	0.80 (< 0.001)	− 0.37 (< 0.001)	− 0.59 (< 0.001)	− 0.14 (0.013)
Social QoL				—	0.73 (< 0.001)	− 0.31 (< 0.001)	− 0.48 (< 0.001)	− 0.12 (0.027)
Environmental QoL					—	− 0.38 (< 0.001)	− 0.47 (< 0.001)	− 0.09 (0.088)
Self‐stigma						—	0.48 (< 0.001)	0.03 (0.569)
Distress							—	0.16 (0.004)
								—

*Note*: # of symptoms indicates number of long COVID symptoms; sleep quality was assessed using the Pittsburgh Sleep Quality Index; QoL was assessed using the World Health Organization Quality of Life Questionnaire‐Brief version; self‐stigma was assessed using the Self‐Stigma Scale‐Short; and psychological distress was assessed using the Depression, Anxiety and Stress Scale‐21.

^a^ Using Spearman's correlation test for correlations with number of symptoms. Other correlations were analyzed using Pearson correlations.

Parallel mediation models showed that long COVID symptoms had significantly direct effects on participants’ poor sleep quality (standardized coefficients [SCs] = 0.38 to 0.39; *p*‐values = 0.002–0.003) and psychological distress (SCs = 0.28–0.29; *p*‐values = 0.023–0.030) but not on self‐stigma (SCs = 0.01–0.02; *p*‐values = 0.906–0.951) and all domains of QoL (SCs = −0.12 to − 0.10; *p*‐values = 0.259–0.455). For instance, the significant direct effect of long COVID symptoms on participants’ (poor) sleep quality with standardized coefficients of 0.38–0.39 indicated that for every one standard deviation increase in long COVID symptoms, there was an equivalent 0.38–0.39 standard deviation increase in poor sleep quality. In addition, poor sleep quality (SCs = −0.26 to − 0.11; *p*‐values < 0.001 to = 0.035), psychological distress (SCs = −0.48 to − 0.34; *p*‐values < 0.001), and self‐stigma (SCs = −0.21 to − 0.11; *p*‐values < 0.001 to = 0.026) had significantly and negatively direct associations with all domains of QoL, except for the association between poor sleep quality and environmental QoL (SC = −0.07; *p* = 0.222). Additionally, mediated effects of (1) poor sleep quality were significant in the associations between long COVID symptoms and physical QoL and social QoL, and (2) psychological distress were significant in the associations between long COVID symptoms and all domains of QoL. Self‐stigma had no mediated effects in the associations between long COVID symptoms and any domain of QoL (Table 5).

**TABLE 5:** Results of parallel mediation models (Hayes’ Process Macro Model 4) reporting mediated effects of sleep quality, psychological distress, and self‐stigma in the association between long COVID symptoms and quality of life (QoL).

Model description Path	Unstandard coefficient (SE)	Standard coefficient	LLCI, ULCI	*p* value
**Dependent variable: physical QoL (*R* ^2^ = 0.44)** **Mediators: sleep quality (*R* ^2^ = 0.07), psychological distress (*R* ^2^ = 0.03), and self‐stigma (*R* ^2^ = 0.01)**				
Long COVID symptoms → sleep quality	1.54 (0.51)	0.38	0.53, 2.55	0.003
Long COVID symptoms → psychological distress	3.57 (1.64)	0.28	0.34, 6.79	0.030
Long COVID symptoms → self‐stigma	0.01 (0.10)	0.02	− 0.18, 0.20	0.906
Sleep quality → physical QoL	− 0.19 (0.03)	− 0.26	− 0.25, − 0.12	< 0.001
Psychological distress → physical QoL	− 0.10 (0.01)	− 0.44	− 0.12, − 0.08	< 0.001
Self‐stigma → physical QoL	− 0.42 (0.18)	− 0.11	− 0.78, − 0.06	0.024
Long COVID symptoms → physical QoL	− 0.33 (0.29)	− 0.11	− 0.89, 0.24	0.259
Long COVID symptoms → sleep quality → physical QoL^a^	− 0.29 (0.11)	− 0.10	− 0.50, − 0.09	—
Long COVID symptoms → psychological distress → physical QoL^a^	− 0.36 (0.18)	− 0.12	− 0.76, − 0.03	—
Long COVID symptoms → self‐stigma → physical QoL^a^	− 0.005 (0.05)	− 0.002	− 0.11, 0.08	—
**Dependent variable: psychological QoL (*R* ^2^ = 0.38)** **Mediators: sleep quality (*R* ^2^ = 0.07), psychological distress (*R* ^2^ = 0.03), and self‐stigma (*R* ^2^ = 0.01)**				
Long COVID symptoms → sleep quality	1.59 (0.52)	0.39	0.58, 2.60	0.002
Long COVID symptoms → psychological distress	3.75 (1.64)	0.29	0.51, 6.98	0.023
Long COVID symptoms → self‐stigma	0.01 (0.10)	0.01	− 0.19, 0.20	0.951
Sleep quality → psychological QoL	− 0.09 (0.04)	− 0.11	− 0.17, − 0.01	0.035
Psychological distress → psychological QoL	− 0.12 (0.01)	− 0.48	− 0.15, − 0.10	< 0.001
Self‐stigma → psychological QoL	− 0.52 (0.22)	− 0.12	− 0.96, − 0.09	0.018
Long COVID symptoms → psychological QoL	− 0.36 (0.35)	− 0.11	− 1.04, 0.32	0.301
Long COVID symptoms → sleep quality → psychological QoL^a^	− 0.14 (0.09)	− 0.04	− 0.35, 0.02	—
Long COVID symptoms → psychological distress → psychological QoL^a^	− 0.47 (0.23)	− 0.14	− 0.96, − 0.06	—
Long COVID symptoms → self‐stigma → psychological QoL^a^	− 0.003 (0.06)	− 0.001	− 0.13, 0.11	—
**Dependent variable: social QoL (*R* ^2^ = 0.26)** **Mediators: sleep quality (*R* ^2^ = 0.07), psychological distress (*R* ^2^ = 0.03), and self‐stigma (*R* ^2^ = 0.01)**				
Long COVID symptoms → sleep quality	1.54 (0.51)	0.38	0.53, 2.55	0.003
Long COVID symptoms → psychological distress	3.57 (1.64)	0.28	0.34, 6.79	0.030
Long COVID symptoms → self‐stigma	0.01 (0.10)	0.02	− 0.18, 0.20	0.906
Sleep quality → social QoL	− 0.10 (0.04)	− 0.13	− 0.18, − 0.02	0.018
Psychological distress → social QoL	− 0.09 (0.01)	− 0.37	− 0.12, − 0.06	< 0.001
Self‐stigma → social QoL	− 0.51 (0.23)	− 0.12	− 0.96, − 0.06	0.026
Long COVID symptoms → social QoL	− 0.27 (0.36)	− 0.09	− 0.97, 0.44	0.455
Long COVID symptoms → sleep quality → social QoL^a^	− 0.16 (0.09)	− 0.05	− 0.36, − 0.001	—
Long COVID symptoms → psychological distress → social QoL^a^	− 0.32 (0.17)	− 0.10	− 0.70, − 0.02	—
Long COVID symptoms → self‐stigma → social QoL^a^	− 0.01 (0.06)	− 0.001	− 0.14, 0.11	—
**Dependent variable: environmental QoL (*R* ^2^ = 0.26)** **Mediators: sleep quality (*R* ^2^ = 0.07), psychological distress (*R* ^2^ = 0.03), and self‐stigma (*R* ^2^ = 0.01)**				
Long COVID symptoms → sleep quality	1.54 (0.51)	0.38	0.53, 2.55	0.003
Long COVID symptoms → psychological distress	3.57 (1.64)	0.28	0.34, 6.79	0.030
Long COVID symptoms → self‐stigma	0.01 (0.10)	0.02	− 0.18, 0.20	0.906
Sleep quality → environmenta QoL	− 0.05 (0.04)	− 0.07	− 0.13, 0.03	0.222
Psychological distress → environmental QoL	− 0.08 (0.01)	− 0.34	− 0.11, − 0.05	< 0.001
Self‐stigma → environmental QoL	− 0.83 (0.22)	− 0.21	− 1.26, − 0.40	< 0.001
Long COVID symptoms → environmental QoL	− 0.29 (0.34)	− 0.10	− 0.96, 0.39	0.401
Long COVID symptoms → sleep quality → environmental QoL^a^	− 0.08 (0.08)	− 0.03	− 0.25, 0.09	—
Long COVID symptoms → psychological distress → environmenta QoL^a^	− 0.28 (0.15)	− 0.10	− 0.61, − 0.02	—
Long COVID symptoms → self‐stigma → environmental QoL^a^	− 0.01 (0.09)	− 0.003	− 0.19, 0.16	—

*Note*: Age and sex were controlled in all mediation models. Sleep quality was assessed using the Pittsburgh Sleep Quality Index; QoL was assessed using the World Health Organization Quality of Life Questionnaire‐Brief version; self‐stigma was assessed using the Self‐Stigma Scale‐Short; psychological distress was assessed using the Depression, Anxiety and Stress Scale‐21.

Abbreviations: ULCI = upper limit confidence interval; LLCI = lower limit confidence interval.

^a^ Mediation tested using bootstrapping method with 5000 bootstrapping resamples.

## 4 Discussion

The present study examined the parallel mediating effects of sleep quality, psychological distress, and self‐stigma in the associations between long COVID symptoms and QoL among Taiwanese individuals with mental health illnesses. Comparison between participants who had long COVID symptoms and those with no symptoms showed that there was no significant difference between the two groups on the study variables except for sleep quality, psychological distress, physical QoL, and psychological QoL. Participants who had long COVID symptoms had poorer sleep quality and greater levels of psychological distress, indicating that they have more sleeping challenges and worsened mental health issues (e.g., depression, anxiety, and stress) as perpetuated by long COVID symptoms compared to those with no symptoms. This suggests that long COVID symptoms may have long‐lasting detrimental mental health issues for individuals with mental health illness, which is similar to other previous studies (e.g., poor sleep quality (Fischer et al. 2022; Bourmistrova et al. 2022; Mekhael et al. 2022) and psychological distress (Fischer et al. 2022; Líška et al. 2022; O' Mahony et al. 2022; Bourmistrova et al. 2022).

Therefore, it is not surprising that participants who had long COVID symptoms had poorer physical and psychological QoL compared to those who did not indicate the enhanced negative effects of long COVID symptoms on their lives. Notwithstanding, the non‐significant between‐group differences on self‐stigma, social QoL, and environmental QoL indicate that in the long term, those with and without long COVID symptoms do not significantly differ on these variables. These findings are quite similar to a previous study that compared individuals with long and short COVID, especially with depression and anxiety (Fancourt, Steptoe, and Bu 2023), which was reflected in the reduction of their physical and mental QoL among those with long COVID (Seeßle et al. 2022). Therefore, proactive measures (e.g., vaccination, relaxation techniques, and wearing of face masks) are needed to prevent or nullify the effects of long COVID symptoms or similar conditions among these individuals.

Correlational analyses showed significant small‐medium positive relationships between sleep quality, psychological distress, and self‐stigma, as well as significant small‐large negative relationships between these three variables and QoL. These relationships were supported by the findings of the mediation models, which showed significant negative direct effects of sleep quality, psychological distress, and self‐stigma on QoL except for sleep quality on environmental QoL. These findings indicate that individuals with higher levels of sleep quality, psychological distress, or self‐stigma will likely have poorer QoL. Moreover, all these findings are consistent with previous studies even before the emergence of COVID‐19 (Marques et al. 2017; Sella et al. 2023).

Mediation analyses showed that long COVID symptoms only indirectly affected physical QoL (via sleep quality or psychological distress), psychological QoL (via psychological distress), social QoL (via sleep quality or psychological distress), and environmental QoL (via psychological distress). These findings indicate that long COVID symptoms may not directly affect an individual's QoL but can still affect an individual's QoL indirectly through psychological distress (and sometimes sleep quality). Possible reasons accounting for the lack of direct effect of long COVID on QoL may include (1) temporal dynamics effect of long COVID on QoL may be longer term and not immediate, more than 1‐month postinfection; (2) social support, environmental factors, and access to healthcare may buffer the direct effects of long COVID; and (3) mediation by intermediate variables (e.g., psychological distress, sleep quality), which may affect QoL (Kim et al. 2023a, 2023b; Lüscher, Scholz, and Bierbauer 2023; Tüzün et al. 2022). Moreover, the findings suggest that sleep quality and/or psychological distress are important variables among individuals with mental health illnesses and are capable of impacting negatively on their QoL. Therefore, clinicians may focus on using therapies such as relaxation and meditation to help enhance sleep quality and psychological state for individuals with mental health illnesses (Hamdani et al. 2022).

Self‐stigma was not a significant mediator, mainly because long COVID symptoms did not directly affect self‐stigma, although self‐stigma affected all the QoL domains suggesting that individuals with mental health illnesses do not stigmatize themselves with long COVID, probably due to their familiarization with COVID‐19. There are no known previous studies examining the mediators of long COVID symptoms and QoL. However, other related studies have found mediating factors such as “social identity affirming behaviors during isolation”, and psychological distress and loneliness in the association between fear of COVID‐19 and QoL (Lardone et al. 2020) and fear of COVID‐19 and life satisfaction (Çağış et al. 2023; Satici et al. 2021), respectively. Therefore, the present study significantly contributes to the knowledge of the impact of long COVID symptoms on individuals’ lives.

### 4.1 Limitations, Strengths, and Implications

The present study had a number of limitations. Firstly, the study used a cross‐sectional survey design, which limited the cause‐and‐effect relationships between the variables despite the robust statistical analysis used. Future studies should use longitudinal methods to establish these cause‐and‐effect relationships. Secondly, there were fewer participants with long COVID symptoms than those without long COVID symptoms, which may have impacted negatively on the statistical analysis, especially the between‐group comparison (Table 2). From the results, it can be deduced that far more significant differences would have been observed if the number of participants were almost the same. Thirdly, participants mainly comprised adults, so the findings may not fully represent child and adolescent cohorts who have long COVID symptoms. Therefore, future studies may consider using children and adolescents to ascertain appropriate information among these cohorts. Fourthly, the majority of the participants were prescribed antipsychotics with different drug action mechanisms (e.g., first or second generation). Therefore, those who were prescribed oral antiviral drugs (i.e., Paxlovid or Molnupiravir) during COVID‐19 infection might have had their antipsychotics temporarily stopped or changed by their psychiatrists to prevent any interaction between the prescribed medications. Consequently, the impacts of antipsychotics might not be strong. However, because of the cessation or changing of the antipsychotics, their psychiatric symptoms might have increased. Fifthly, the study used participants who had symptoms of COVID‐19 after 1‐month postinfection, which is arguably borderline for long COVID diagnosis. Therefore, this may have increased the number of participants with a long COVID for the study (Durstenfeld et al. 2023). Lastly, the study only used patients who visited the center (JPC) and not homecare patients. This was because there were only a few physicians trained to evaluate COVID‐19 symptoms, and these physicians were not part of the homecare service personnel. Therefore, caution is needed in generalizing the findings to homecare patients.

Despite these limitations, the present study had novel findings in terms of the mediating factors in the association between long COVID symptoms and QoL. The findings provided novel information on the indirect pathways through which long COVID symptoms may affect the QoL among individuals with mental health illnesses. Consequently, the data suggest that individuals with long COVID symptoms will likely have poor QoL due to their poor sleep quality or greater psychological distress brought about by long COVID. Therefore, it is recommended that individuals with mental health illnesses should be taught relaxation and meditation techniques in addition to psychoeducation on (long) COVID to help stem the negative effect on their lives.

## 5 Conclusion

The present study was novel and examined the parallel mediating effects of sleep quality, psychological distress, and self‐stigma in the associations between long COVID symptoms and QoL. It showed that although there was no direct effect of long COVID symptoms on QoL, there were other alternative pathways for long COVID symptoms to affect individuals’ QoL. This was mainly through psychological distress and sometimes through sleep quality. These findings suggest that individuals with long COVID symptoms have a higher chance of having poor QoL, and so there may be the need for counseling and possible therapy for those who contract COVID‐19, especially among individuals who already have mental health illnesses.

## Author Contributions

**Kun‐Chia Chang**: conceptualization, methodology, data curation, resources, writing–original draft, writing–review and editing, validation, investigation, funding acquisition. **Daniel Kwasi Ahorsu**: conceptualization, methodology, writing–original draft, writing–review and editing. **Hsin‐Chi Tsai**: conceptualization, methodology, writing–review and editing, validation, funding acquisition, investigation. **Carol Strong**: conceptualization, methodology, writing–review and editing. **Nai‐Ying Ko**: conceptualization, methodology, writing–review and editing. **Jung‐Sheng Chen**: conceptualization, methodology, writing–review and editing. **Cheng‐Fang Yen**: conceptualization, methodology, writing–review and editing. **Servet Üztemur**: conceptualization, methodology, validation, writing–original draft, writing–review and editing, funding acquisition. **Mark D. Griffiths**: methodology, writing–review and editing. **Chung‐Ying Lin**: conceptualization, methodology, validation, visualization, investigation, funding acquisition, writing–original draft, writing–review and editing, supervision, software, formal analysis, project administration.

### Peer Review

The peer review history for this article is available at https://publons.com/publon/10.1002/brb3.70094.

## Data Availability

The data that support the findings of this study are available on request from the corresponding author. The data are not publicly available due to privacy or ethical restrictions.

## Endnotes

1 The Taiwan government classifies infectious diseases into five categories. Individuals who are identified to be infected by a Category 1 infectious disease need to be reported to the authorities within 24 h and receive intervention in quarantine. Individuals who are identified to be infected by a Category 2 infectious disease need to be reported to the authorities within 24 h and receive intervention in quarantine if necessary. Individuals who are identified to be infected by a Category 3 infectious disease need to be reported to the authorities within 1 week and receive intervention in quarantine if necessary. Individuals who are identified to be infected by a Category 4 infectious disease need to be reported to the authorities. Category 5 infectious diseases are not classified in Categories 1–4, but the authorities need to be prepared for prevention.

## References

[brb370094-bib-0001] Addo, I. Y. 2020. “Double Pandemic: Racial Discrimination Amid Coronavirus Disease 2019.” Social Sciences & Humanities Open 2, no. 1: 100074. 10.1016/j.ssaho.2020.100074. 34173502 PMC7574839

[brb370094-bib-0002] Aghaei, A. , R. Zhang , S. Taylor , et al. 2022. “Impact of COVID‐19 Symptoms on Social Aspects of Life Among Female Long Haulers: A Qualitative Study.” Research Square rs.3.rs–1285284. 10.21203/rs.3.rs-1285284/v1.

[brb370094-bib-0003] Al‐Aly, Z. , B. Bowe , and Y. Xie . 2022. “Long COVID After Breakthrough SARS‐CoV‐2 Infection.” Nature Medicine 28, no. 7: 1461–1467. 10.1038/s41591-022-01840-0. PMC9307472 35614233

[brb370094-bib-0004] Alimohamadi, Y. , M. Sepandi , M. Taghdir , and H. Hosamirudsari . 2020. “Determine the Most Common Clinical Symptoms in COVID‐19 Patients: A Systematic Review and Meta‐analysis.” The Journal of Preventive Medicine and Hygiene 61, no. 3: E304–E312. 10.15167/2421-4248/jpmh2020.61.3.1530. 33150219 PMC7595075

[brb370094-bib-0005] AlNujaidi, H. , A. Alfayez , A. Alsaif , D. Alsalman , S. Almubarak , and S. Almulla . 2022. “The Influence of Partial Curfew on the Quality of Life in the Kingdom of Saudi Arabia During the COVID‐19 Pandemic.” Open Public Health Journal 15: e187494452207130. 10.2174/18749445-v15-e2207130.

[brb370094-bib-0006] Andrade, C. , M. Gillen , J. A. Molina , and M. J. Wilmarth . 2022. “The Social and Economic Impact of Covid‐19 on Family Functioning and Well‐Being: Where Do We Go From Here?” Journal of Family and Economic Issues 43, no. 2: 205–212. 10.1007/s10834-022-09848-x. 35669394 PMC9136200

[brb370094-bib-0007] Ashwlayan, V. D. , C. Antlash , M. Imran , et al. 2022. “Insight Into the Biological Impact of COVID‐19 and Its Vaccines on Human Health.” Saudi Journal of Biological Sciences 29, no. 5: 3326–3337. 10.1016/j.sjbs.2022.02.010. 35185356 PMC8837491

[brb370094-bib-0008] Atashi, V. , S. Mohammadi , Z. Salehi , Z. Shafiei , M. Savabi‐Esfahani , and K. Salehi . 2023. “Challenges Related to Health Care for Iranian Women With Breast Cancer During the COVID‐19 Pandemic: A Qualitative Study.” Asian Journal of Social Health and Behavior 6, no. 2: 72–78. 10.4103/shb.shb_205_22.

[brb370094-bib-0009] Ballering, A. , T. Olde Hartman , and J. Rosmalen . 2021. “Long COVID‐19, Persistent Somatic Symptoms and Social Stigmatisation.” Journal of Epidemiology and Community Health 75, no. 6: 603–604. 10.1136/jech-2021-216643. 33622802 PMC8142431

[brb370094-bib-0010] Beyerstedt, S. , E. B. Casaro , and É. B. Rangel . 2021. “COVID‐19: Angiotensin‐Converting Enzyme 2 (ACE2) Expression and Tissue Susceptibility to SARS‐CoV‐2 Infection.” European Journal of Clinical Microbiology & Infectious Diseases 40, no. 5: 905–919. 10.1007/s10096-020-04138-6. 33389262 PMC7778857

[brb370094-bib-0011] Bourmistrova, N. W. , T. Solomon , P. Braude , R. Strawbridge , and B. Carter . 2022. “Long‐Term Effects of COVID‐19 on Mental Health: A Systematic Review.” Journal of Affective Disorders 299: 118–125. 10.1016/j.jad.2021.11.031. 34798148 PMC8758130

[brb370094-bib-0012] Brooks, S. K. , R. K. Webster , L. E. Smith , et al. 2020. “The Psychological Impact of Quarantine and How to Reduce It: Rapid Review of the Evidence.” Lancet 395, no. 10227: 912–920. 10.1016/S0140-6736(20)30460-8. 32112714 PMC7158942

[brb370094-bib-0013] Brooks‐Hay, O. , K. Saunders , and M. Burman . 2022. “A Toxic Mix: The Impact of COVID‐19 Lockdown Measures on the Post‐Separation Experiences of Domestic Abuse Survivors.” Journal of Gender‐Based Violence 6, no. 3: 426–441. 10.1332/239868021x16536613142067.

[brb370094-bib-0014] Buysse, D. J. , C. F. Reynolds , T. H. Monk , S. R. Berman , and D. J. Kupfer . 1989. “The Pittsburgh Sleep Quality Index: A New Instrument for Psychiatric Practice and Research.” Psychiatry Research 28, no. 2: 193–213. 10.1016/0165-1781(89)90047-4. 2748771

[brb370094-bib-0015] Byambasuren, O. , P. Stehlik , J. Clark , K. Alcorn , and P. Glasziou . 2023. “Effect of COVID‐19 Vaccination on Long Covid: Systematic Review.” BMJ Medicine 2, no. 1:e000385. 10.1136/bmjmed-2022-000385. 36936268 PMC9978692

[brb370094-bib-0016] Çağış, Z. G. , G. G. Öztekin , I. A. Aziz , F. Chirico , A. Rizzo , and M. Yıldırım . 2023. “Meaning in Life and Loneliness as Mediators Between COVID‐19 Anxiety and Life Satisfaction in the Post‐Pandemic Among the General Population in Turkey: A Serial Mediation Model.” European Journal of Investigation in Health, Psychology and Education 13, no. 10: 2214–2225. 10.3390/ejihpe13100156. 37887157 PMC10606174

[brb370094-bib-0017] Cao, C. , X. Liao , X. Jiang , X. Li , I. Chen , and C. Lin . 2023. “Psychometric Evaluation of the Depression, Anxiety, and Stress Scale‐21 (DASS‐21) Among Chinese Primary and Middle School Teachers.” BMC Psychology 11, no. 1: 209. 10.1186/s40359-023-01242-y. 37452365 PMC10349442

[brb370094-bib-0018] Carpenter, J. S. , and M. A. Andrykowski . 1998. “Psychometric Evaluation of the Pittsburgh Sleep Quality Index.” Journal of Psychosomatic Research 45, no. 1: 5–13. 10.1016/s0022-3999(97)00298-5. 9720850

[brb370094-bib-0019] Ceban, F. , S. Ling , L. M. W. Lui , et al. 2022. “Fatigue and Cognitive Impairment in Post‐COVID‐19 Syndrome: A Systematic Review and Meta‐Analysis.” Brain, Behavior, and Immunity 101: 93–135. 10.1016/j.bbi.2021.12.020. 34973396 PMC8715665

[brb370094-bib-0020] Chang, K. , W. Hou , A. H. Pakpour , C. Lin , and M. D. Griffiths . 2022. “Psychometric Testing of Three COVID‐19‐related Scales Among People With Mental Illness.” International Journal of Mental Health and Addiction 20, no. 1: 324–336. 10.1007/s11469-020-00361-6. 32837442 PMC7354353

[brb370094-bib-0021] Chang, K. , C. Strong , A. H. Pakpour , M. D. Griffiths , and C. Lin . 2020. “Factors Related to Preventive COVID‐19 Infection Behaviors Among People With Mental Illness.” Journal of the Formosan Medical Association 119, no. 12: 1772–1780. 10.1016/j.jfma.2020.07.032. 32773260 PMC7388748

[brb370094-bib-0022] Chen, I. , C. Chen , X. Liao , et al. 2023. “Psychometric Properties of the Depression, Anxiety, and Stress Scale (DASS‐21) Among Different Chinese Populations: A Cross‐Sectional and Longitudinal Analysis.” Acta Psychologica 240: 104042. 10.1016/j.actpsy.2023.104042. 37783184

[brb370094-bib-0023] Dale, R. , S. Budimir , T. Probst , E. Humer , and C. Pieh . 2022. “Quality of Life During the COVID‐19 Pandemic in Austria.” Frontiers in Psychology 13: 934253. 10.3389/fpsyg.2022.934253. 35978783 PMC9376461

[brb370094-bib-0024] Davis, H. E. , G. S. Assaf , L. McCorkell , et al. 2021. “Characterizing Long COVID in an International Cohort: 7 Months of Symptoms and Their Impact.” eClinicalMedicine 38: 101019. 10.1016/j.eclinm.2021.101019. 34308300 PMC8280690

[brb370094-bib-0025] Davis, H. E. , L. McCorkell , J. M. Vogel , and E. J. Topol . 2023. “Long COVID: Major Findings, Mechanisms and Recommendations.” Nature Reviews Microbiology 21, no. 3: 133–146. 10.1038/s41579-022-00846-2. 36639608 PMC9839201

[brb370094-bib-0026] Druss, B. G. 2020. “Addressing the COVID‐19 Pandemic in Populations With Serious Mental Illness.” JAMA Psychiatry 77, no. 9: 891–892. 10.1001/jamapsychiatry.2020.0894. 32242888

[brb370094-bib-0027] Durstenfeld, M. S. , M. J. Peluso , N. D. Peyser , et al. 2023. “Factors Associated With Long COVID Symptoms in an Online Cohort Study.” Open Forum Infectious Diseases 10, no. 2:ofad047. 10.1093/ofid/ofad047. 36846611 PMC9945931

[brb370094-bib-0028] Fancourt, D. , A. Steptoe , and F. Bu . 2023. “Psychological Consequences of Long COVID: Comparing Trajectories of Depressive and Anxiety Symptoms Before and After Contracting SARS‐CoV‐2 Between Matched Long‐ and Short‐COVID Groups.” British Journal of Psychiatry 222, no. 2: 74–81. 10.1192/bjp.2022.155. PMC7614126 36458509

[brb370094-bib-0029] Figueiredo, M.dC. , J. Amendoeira , M. Rosa , R. Matos , M. Silva , and L. Gonzaga . 2021. “Quality of Life of Students in Polytechnic Higher Education at the Santarem and Leiria: The Impact of COVID‐19.” European Journal of Public Health 31, no. S2: ckab120.036. 10.1093/eurpub/ckab120.036.

[brb370094-bib-0030] Fischer, A. , L. Zhang , A. Elbéji , et al. 2022. “Long COVID Symptomatology After 12 Months and Its Impact on Quality of Life According to Initial Coronavirus Disease 2019 Disease Severity.” Open Forum Infectious Diseases 9, no. 8: ofac397. 10.1093/ofid/ofac397. 35983269 PMC9379809

[brb370094-bib-0031] Grant, M. C. , L. Geoghegan , M. Arbyn , et al. 2020. “The Prevalence of Symptoms in 24,410 Adults Infected by the Novel Coronavirus (SARS‐CoV‐2; COVID‐19): A Systematic Review and Meta‐Analysis of 148 Studies From 9 Countries.” PLoS ONE 15, no. 6: e0234765. 10.1371/journal.pone.0234765. 32574165 PMC7310678

[brb370094-bib-0032] Hamdani, S. U. , S. W. Zafar , N. Suleman , A. Waqas , and A. Rahman . 2022. “Effectiveness of Relaxation Techniques ‘as an Active Ingredient of Psychological Interventions’ to Reduce Distress, Anxiety and Depression in Adolescents: A Systematic Review and Meta‐Analysis.” International Journal of Mental Health Systems 16, no. 1: 31. 10.1186/s13033-022-00541-y. 35765083 PMC9238062

[brb370094-bib-0033] Hawlader, M. D. H. , M. U. Rashid , M. A. S. Khan , et al. 2021. “Quality of Life of COVID‐19 Recovered Patients in Bangladesh.” PLoS ONE 16, no. 10: e0257421. 10.1371/journal.pone.0257421. 34644332 PMC8513834

[brb370094-bib-0034] Hossain, M. M. , A. Sultana , and N. Purohit . 2020. “Mental Health Outcomes of Quarantine and Isolation for Infection Prevention: A Systematic Umbrella Review of the Global Evidence.” Epidemiology Health 42: e2020038. 10.4178/epih.e2020038. 32512661 PMC7644933

[brb370094-bib-0035] Hwang, H. , W. Liang , Y. Chiu , and M. Lin . 2003. “Suitability of the WHOQOL‐BREF for Community‐Dwelling Older People in Taiwan.” Age and Ageing 32, no. 6: 593–600. 10.1093/ageing/afg102. 14599999

[brb370094-bib-0036] Kar, B. , N. Kar , and M. C. Panda . 2023. “Social Trust and COVID‐Appropriate Behavior: Learning From the Pandemic.” Asian Journal of Social Health and Behavior 6, no. 3: 93–104. 10.4103/shb.shb_183_22.

[brb370094-bib-0037] Khankeh, H. , M. Pourebrahimi , M. F. Karibozorg , et al. 2022. “Public Trust, Preparedness, and the Influencing Factors Regarding COVID‐19 Pandemic Situation in Iran: A Population‐Based Cross‐Sectional Study.” Asian Journal of Social Health and Behavior 5, no. 4: 154–161. 10.4103/shb.shb_155_22.

[brb370094-bib-0038] Kim, Y. , S. Bae , H. Chang , and S. Kim . 2024. “Characteristics of Long COVID and the Impact of COVID‐19 Vaccination on Long COVID 2 Years Following COVID‐19 Infection: Prospective Cohort Study.” Scientific Reports 14, no. 1: 854. 10.1038/s41598-023-50024-4. 38191556 PMC10774352

[brb370094-bib-0039] Kim, Y. , S. Bae , H. H. Chang , and S. W. Kim . 2023a. “Long COVID Prevalence and Impact on Quality of Life 2 Years After Acute COVID‐19.” Scientific Reports 13, no. 1: 11207. 10.1038/s41598-023-36995-4. 37433819 PMC10336045

[brb370094-bib-0040] Kim, Y. , S. Bae , H. H. Chang , and S. W. Kim . 2023b. “Long COVID Prevalence and Impact on Quality of Life 2 Years After Acute COVID‐19.” Erratum In: Scientific Reports 13, no. 1: 11960. 10.1038/s41598-023-39132-3. 37488240 PMC10366175

[brb370094-bib-0041] Kumar, A. , R. K. Narayan , P. Prasoon , et al. 2021. “COVID‐19 Mechanisms in the Human Body—What We Know so Far.” Frontiers in Immunology 12: 693938. 10.3389/fimmu.2021.693938. 34790191 PMC8592035

[brb370094-bib-0042] Lardone, A. , P. Sorrentino , F. Giancamilli , et al. 2020. “Psychosocial Variables and Quality of Life During the COVID‐19 Lockdown: A Correlational Study on a Convenience Sample of Young Italians.” PeerJ 8: e10611. 10.7717/peerj.10611. 33384910 PMC7751426

[brb370094-bib-0043] Líška, D. , E. Liptaková , A. Babičová , L. Batalik , P. S. Baňárová , and S. Dobrodenková . 2022. “What Is the Quality of Life in Patients With Long COVID Compared to a Healthy Control Group?” Frontiers of Public Health 10: 975992. 10.3389/fpubh.2022.975992. PMC9667067 36408018

[brb370094-bib-0044] Loades, M. E. , E. Chatburn , N. Higson‐Sweeney , et al. 2020. “Rapid Systematic Review: The Impact of Social Isolation and Loneliness on the Mental Health of Children and Adolescents in the Context of COVID‐19.” Journal of the American Academy of Child and Adolescent Psychiatry 59, no. 11: 1218–1239. 10.1016/j.jaac.2020.05.009. 32504808 PMC7267797

[brb370094-bib-0045] Lopez‐Leon, S. , T. Wegman‐Ostrosky , C. Perelman , et al. 2021. “More Than 50 Long‐Term Effects of COVID‐19: A Systematic Review and Meta‐Analysis.” Scientific Reports 11, no. 1: 16144. 10.1038/s41598-021-95565-8. 34373540 PMC8352980

[brb370094-bib-0046] Lovibond, P. F. , and S. H. Lovibond . 1995. “The Structure of Negative Emotional States: Comparison of the Depression Anxiety Stress Scales (DASS) With the Beck Depression and Anxiety Inventories.” Behaviour Research and Therapy 33, no. 3: 335–343. 10.1016/0005-7967(94)00075-u. 7726811

[brb370094-bib-0047] Lüscher, J. , U. Scholz , and W. Bierbauer . 2023. “Social Support, Distress and Well‐Being in Individuals Experiencing Long‐COVID: A Cross‐Sectional Survey Study.” BMJ Open 13, no. 3:e067166. 10.1136/bmjopen-2022-067166. PMC10039976 36948566

[brb370094-bib-0048] Mahlangu, P. , A. Gibbs , N. Shai , M. Machisa , N. Nunze , and Y. Sikweyiya . 2022. “Impact of COVID‐19 Lockdown and Link to Women and Children's Experiences of Violence in the Home in South Africa.” BMC Public Health 22, no. 1: 1029. 10.1186/s12889-022-13422-3. 35597933 PMC9123923

[brb370094-bib-0049] Mak, W. W. S. , and R. Y. M. Cheung . 2010. “Self‐Stigma Among Concealable Minorities in Hong Kong: Conceptualization and Unified Measurement.” American Journal of Orthopsychiatry 80, no. 2: 267–281. 10.1111/j.1939-0025.2010.01030.x. 20553520

[brb370094-bib-0050] Marques, D. R. , A. M. S. Meia‐Via , C. F. da Silva , and A. A. Gomes . 2017. “Associations Between Sleep Quality and Domains of Quality of Life in a Non‐Clinical Sample: Results From Higher Education Students.” Sleep Health 3, no. 5: 348–356. 10.1016/j.sleh.2017.07.004. 28923191

[brb370094-bib-0051] Mekhael, M. , C. H. Lim , A. H. El Hajjar , et al. 2022. “Studying the Effect of Long COVID‐19 Infection on Sleep Quality Using Wearable Health Devices: Observational Study.” Journal of Medical Internet Research 24, no. 7: e38000. 10.2196/38000. 35731968 PMC9258734

[brb370094-bib-0052] Mouratidis, K. , and A. Yiannakou . 2022. “COVID‐19 and Urban Planning: Built Environment, Health, and Well‐Being in Greek Cities Before and During the Pandemic.” Cities 121: 103491. 10.1016/j.cities.2021.103491. 34658478 PMC8501234

[brb370094-bib-0053] Murphy, L. , K. Markey , C. O' Donnell , M. Moloney , and O. Doody . 2021. “The Impact of the COVID‐19 Pandemic and Its Related Restrictions on People With Pre‐Existent Mental Health Conditions: A Scoping Review.” Archives of Psychiatric Nursing 35, no. 4: 375–394. 10.1016/j.apnu.2021.05.002. 34176579 PMC9759111

[brb370094-bib-0054] O' Mahony, L. , T. Buwalda , M. Blair , et al. 2022. “Impact of Long COVID on Health and Quality of Life.” HRB Open Research 5: 31. 10.12688/hrbopenres.13516.1. 36101871 PMC9440374

[brb370094-bib-0055] Orrù, G. , D. Bertelloni , F. Diolaiuti , et al. 2021. “Long‐COVID Syndrome? A Study on the Persistence of Neurological, Psychological and Physiological Symptoms.” Healthcare 9, no. 5: 575. 10.3390/healthcare9050575. 34068009 PMC8152255

[brb370094-bib-0056] Pantelic, M. , N. Ziauddeen , M. Boyes , M. E. O'Hara , C. Hastie , and N. A. Alwan . 2022. “Long Covid Stigma: Estimating Burden and Validating Scale in a UK‐Based Sample.” PLoS ONE 17, no. 11: e0277317. 10.1371/journal.pone.0277317. 36417364 PMC9683629

[brb370094-bib-0057] Poudel, A. N. , S. Zhu , N. Cooper , et al. 2021. “Impact of Covid‐19 on Health‐Related Quality of Life of Patients: A Structured Review.” PLoS ONE 16, no. 10: e0259164. 10.1371/journal.pone.0259164. 34710173 PMC8553121

[brb370094-bib-0058] Saffari, M. , K. Chang , J. Chen , et al. 2023. “Sleep Quality and Self‐Stigma Mediate the Association Between Problematic Use of Social Media and Quality of Life Among People With Schizophrenia in Taiwan: A Longitudinal Study.” Psychiatry Investigation 20, no. 11: 1034–1044. 10.30773/pi.2023.0169. 37997331 PMC10678148

[brb370094-bib-0059] Satici, B. , E. Gocet‐Tekin , M. E. Deniz , and S. A. Satici . 2021. “Adaptation of the Fear of COVID‐19 Scale: Its Association With Psychological Distress and Life Satisfaction in Turkey.” International Journal of Mental Health and Addiction 19, no. 6: 1980–1988. 10.1007/s11469-020-00294-0. 32395095 PMC7207987

[brb370094-bib-0060] Scholz, U. , W. Bierbauer , and J. Lüscher . 2023. “Social Stigma, Mental Health, Stress, and Health‐Related Quality of Life in People With Long COVID.” International Journal of Environmental Research and Public Health 20, no. 5: 3927. 10.3390/ijerph20053927. 36900938 PMC10001775

[brb370094-bib-0061] Seeßle, J. , T. Waterboer , T. Hippchen , et al. 2022. “Persistent Symptoms in Adult Patients 1 Year After Coronavirus Disease 2019 (COVID‐19): A Prospective Cohort Study.” Clinical Infectious Diseases 74, no. 7: 1191–1198. 10.1093/cid/ciab611. 34223884 PMC8394862

[brb370094-bib-0062] Sella, E. , L. Miola , E. Toffalini , and E. Borella . 2023. “The Relationship Between Sleep Quality and Quality of Life in Aging: A Systematic Review and Meta‐Analysis.” Health Psychology Review 17, no. 1: 169–191. 10.1080/17437199.2021.1974309. 34459704

[brb370094-bib-0063] Shen, Q. , E. E. Joyce , O. V. Ebrahimi , et al. 2023. “COVID‐19 Illness Severity and 2‐Year Prevalence of Physical Symptoms: An Observational Study in Iceland, Sweden, Norway and Denmark.” Lancet Regional Health—Europe 35: 100756. 10.1016/j.lanepe.2023.100756. 38115966 PMC10730314

[brb370094-bib-0064] Sivan, M. , and S. Taylor . 2020. “NICE Guideline on Long Covid.” BMJ 371: m4938. 10.1136/bmj.m4938. 33361141

[brb370094-bib-0065] Subramanian, A. , K. Nirantharakumar , S. Hughes , et al. 2022. “Symptoms and Risk Factors for Long COVID in Non‐Hospitalized Adults.” Nature Medicine 28, no. 8: 1706–1714. 10.1038/s41591-022-01909-w. PMC9388369 35879616

[brb370094-bib-0066] THE WHOQOL GROUP . 1998. “Development of the World Health Organization WHOQOL‐BREF Quality of Life Assessment.” Psychological Medicine 28, no. 3: 551–558. 10.1017/S0033291798006667. 9626712

[brb370094-bib-0067] Trihandini, I. , M. Muhtar , D. A. Karunia Sakti , and C. P. Erlianti . 2023. “The Effect of Long‐Haul COVID‐19 Toward Domains of the Health‐Related Quality of Life Among Recovered Hospitalized Patients.” Frontiers in Public Health 11: 1068127. 10.3389/fpubh.2023.1068127. 37601220 PMC10434763

[brb370094-bib-0068] Tsai, P. , S. Wang , M. Wang , et al. 2005. “Psychometric Evaluation of the Chinese Version of the Pittsburgh Sleep Quality Index (CPSQI) in Primary Insomnia and Control Subjects.” Quality of Life Research 14, no. 8: 1943–1952. 10.1007/s11136-005-4346-x. 16155782

[brb370094-bib-0069] Tüzün, H. , C. Özbaş , B. Budak , G. Altunay , and F. B. Aksakal . 2022. “Patterns in the Relationship Between Acute COVID‐19/Long COVID‐19 and Quality of Life: A Cross‐Sectional Study of Patients Attending a Tertiary Care Hospital in Turkey.” Asian Pacific Journal of Tropical Medicine 15, no. 6: 274–282. 10.4103/1995-7645.345943.

[brb370094-bib-0070] Vicerra, P. M. M. 2022. “Mental Stress and Well‐Being Among Low‐Income Older Adults During COVID‐19 Pandemic.” Asian Journal of Social Health and Behavior 5, no. 3: 101–107. 10.4103/shb.shb_110_22.

[brb370094-bib-0071] Vicerra, P. M. M. , J. C. G. De Pano , and J. M. Estanislao . 2022. “Nutrition Status of Lower‐Income Older Adults in Thailand During COVID‐19 Pandemic.” Asian Journal of Social Health and Behavior 5, no. 4: 147–153. 10.4103/shb.shb_150_22.

[brb370094-bib-0072] Wang, L. , Y. Wu , Y. Lin , et al. 2022. “Reliability and Validity of the Pittsburgh Sleep Quality Index Among Frontline COVID‐19 Health Care Workers Using Classical Test Theory and Item Response Theory.” Journal of Clinical Sleep Medicine 18, no. 2: 541–551. 10.5664/jcsm.9658. 34534069 PMC8805004

[brb370094-bib-0073] World Health Organization . 2021. A Clinical Case Definition of Post COVID‐19 Condition by a Delphi Consensus. Accessed January 26, 2024. https://www.who.int/publications/i/item/WHO‐2019‐nCoV‐Post_COVID‐19_condition‐Clinical_case_definition‐2021.1.

[brb370094-bib-0074] Wu, T. , C. Chang , C. Chen , J. Wang , and C. Lin . 2015. “Further Psychometric Evaluation of the Self‐Stigma Scale‐Short: Measurement Invariance Across Mental Illness and Gender.” PLoS ONE 10, no. 2: e0117592. 10.1371/journal.pone.0117592. 25659115 PMC4320062

[brb370094-bib-0075] Yao, G. , C. Chung , C. Yu , and J. Wang . 2002. “Development and Verification of Validity and Reliability of the WHOQOL‐BREF Taiwan Version.” Journal of the Formosan Medical Association 101, no. 5: 342–351. 12101852

[brb370094-bib-0076] Yao, H. , J. Chen , and Y. Xu . 2020. “Patients With Mental Health Disorders in the COVID‐19 Epidemic.” Lancet Psychiatry 7, no. 4: e21. 10.1016/S2215-0366(20)30090-0. 32199510 PMC7269717

[brb370094-bib-0077] Yen, C. , C. Chen , Y. Lee , T. Tang , C. Ko , and J. Yen . 2009. “Association Between Quality of Life and Self‐Stigma, Insight, and Adverse Effects of Medication in Patients With Depressive Disorders.” Depression and Anxiety 26, no. 11: 1033–1039. 10.1002/da.20413. 19288581

[brb370094-bib-0078] Zheng, S. , R. Wang , S. Zhang , et al. 2023. “Depression Severity Mediates Stigma and Quality of Life in Clinically Stable People With Schizophrenia in Rural China.” BMC Psychiatry 23, no. 1: 826. 10.1186/s12888-023-05355-x. 37951892 PMC10640747

[brb370094-bib-0079] Zhu, B. , M. Xie , C. G. Park , and M. C. Kapella . 2018. “Adaptation of the Pittsburgh Sleep Quality Index in Chinese Adults With Type 2 Diabetes.” Journal of the Chinese Medical Association 81, no. 3: 242–247. 10.1016/j.jcma.2017.06.021. 29258729 PMC6873698

